# Abstract : stomp in HPFT

**DOI:** 10.1192/bjo.2021.587

**Published:** 2021-06-18

**Authors:** Anu Sharma, Kamalika Mukherji, Chetan Shah

**Affiliations:** 1 Saffron Ground, Ditchmore lane; 2 Colonades; 3 Kingfisher Court

## Abstract

**Aims:**

Analyse the pattern of psychotropic drug use and deprescribing (in the context of STOMP) in people with Intellectual disability and Challenging behaviour in Hertfordshire community team(s) during 2016-17. STOMP stands for Stopping Over Medication in People with Learning Disability, Autism or both.

**Background:**

Public Health England in 2015 estimated that on an average day in England, between 30,000 and 35,000 adults with a learning disability, autism or both were taking prescribed psychotropics without appropriate clinical indications . HPFT signed up to the STOMP pledge in 2017 to actively review psychotropic prescribing in line with NICE guidance alongside patients, carers and professional partnerships. This audit provides the outcomes of applying the STOMP Pledge to clinical practice.

**Method:**

Data collection for the current audit occurred over Q1-5 in 2016–2017. All patients with Intellectual Disabilities on psychotropic medication were reviewed in psychiatric clinics. Awareness was raised about STOMP in teams. A semi-structured tool was developed based on the Self assessment framework published by the ID faculty RCPsych and prospective data were collected after each outpatient visit.

**Result:**

347 patients were prescribed psychotropic medication and reviewed quarterly between 2016-2017. 96 patients were prescribed antipsychotics for challenging behaviour. Other prescribed medications included mood stabilisers, anticonvulsants, anti-depressants and benzodiazepines. Common antipsychotics used: Risperidone (63), Aripiprazole (14), Quetiapine (9), Olanzapine (4); Chlorpromazine (2). Four patients were maintained on two antipsychotics in varying combinations. The data collection tool noted that alternatives to medication were tried in 32 cases. Deprescribing occurred in 41 cases

**Conclusion:**

This study represents an attempt to capture the impact of the STOMP principles in a clinical sample. Various alternatives to medications were pursued in the sample such as positive behaviour support, sensory integration, psychological therapies, social support. Younger adults (under 30 years) represented the largest proportion of cases where medication was increased. Adults over 30 years represented the largest proportion of cases where a STOMP reduction occurred. This may reflect the individual factors at play. Younger people with ID and /or Autism are more likely to experience changes in support and structure at transition, whilst older adults may have more physical comorbidities that may influence this decision.zcvv

